# The systemic inflammation markers as potential predictors of disease progression and survival time in amyotrophic lateral sclerosis

**DOI:** 10.3389/fnins.2025.1552949

**Published:** 2025-03-05

**Authors:** Ye Hong, Jian-Quan Shi, Shuai Feng, Shi-Qi Huang, Zhen-Hua Yuan, Shen Liu, Xiao-Hao Zhang, Jun-Shan Zhou, Teng Jiang, Hong-Dong Zhao, Ying-Dong Zhang

**Affiliations:** Department of Neurology, Nanjing First Hospital, Nanjing Medical University, Nanjing, Jiangsu, China

**Keywords:** amyotrophic lateral sclerosis, systemic inflammation markers, neutrophil to lymphocyte ratio, lymphocyte to monocyte ratio, disease progression rate, survival

## Abstract

**Introduction:**

Amyotrophic lateral sclerosis (ALS) is a fatal and untreatable neurodegenerative disease with only 3–5 years' survival time after diagnosis. Inflammation has been proven to play important roles in ALS progression. However, the relationship between systemic inflammation markers and ALS has not been well established, especially in Chinese ALS patients. The present study aimed to assess the predictive value of systemic inflammation markers including neutrophil to lymphocyte ratio (NLR), platelet to lymphocyte ratio (PLR), lymphocyte to monocyte ratio (LMR), and systemic immune-inflammation index (SII) for Chinese amyotrophic lateral sclerosis (ALS).

**Methods:**

Seventy-two Chinese ALS patients and 73 controls were included in this study. The rate of disease progression was calculated as the change of Revised ALS Functional Rating Scale (ALSFRS-R) score per month. Patients were classified into fast progressors if the progression rate > 1.0 point/month and slow progressors if progression rate ≤ 1.0 point/month. The value of NLR, PLR, LMR, and SII were measured based on blood cell counts. The association between systemic inflammation markers and disease progression rate was confirmed by logistic regression analysis. Kaplan-Meier curve and Cox regression models were used to evaluate factors affecting the survival outcome of ALS patients.

**Results:**

For Chinese ALS patients, NLR, PLR and SII were higher, LMR was lower when compared with controls. All these four markers were proved to be independent correlated with fast progression of ALS. Both Kaplan-Meier curve and Cox regression analysis indicated that higher NLR and lower LMR were associated with shorter survival time in the ALS patients.

**Discussion:**

In conclusion, the systemic inflammation markers, especially NLR and LMR might be independent markers for rapid progression and shorter survival time in Chinese ALS patients.

## 1 Introduction

Amyotrophic lateral sclerosis (ALS) is a relentlessly progressive and fatal neurodegenerative disease, which selectively affects motor neurons, leading to progressive muscle atrophy and limb weakness, dysphagia, and eventually to paralysis and respiratory failure (Al-Chalabi and Hardiman, 2013). Most of ALS patients die within 3–5 years after the onset of symptoms (van Es et al., 2017). Although ALS is relatively rare, affecting 1–1.8/100,000 individuals worldwide, the number of ALS cases are expected to increase from 222,801 in 2015 to 376,674 in 2040 due to the aging of the global population, leading to huge burden to families and society (Arthur et al., 2016). However, there is no effective therapy to delay or stop the onset or progression of ALS as the pathogenesis of ALS remains elusive. Identification of inexpensive and easy-to-implement biomarkers of ALS which could predict disease progression rate and survival time is of great importance for improving disease management.

Neuroinflammation and peripheral immunity have been postulated to play important roles in the neurodegenerative process of ALS (Liu et al., 2020). Neuroinflammation is always induced by microglia and astrocyte activation, which could be detected by the immunostaining of the post mortem tissue from ALS patients (Kawamata et al., 1992; Schiffer et al., 1996). The advances in positron emission tomography (PET) imaging made it possible to visualize active gliosis in humans *in vivo* through coupled proteins expressed by activated microglia (Corcia et al., 2012). However, above methods to evaluate neuroinflammation are invasive, costly and technically difficult, which cannot be implemented in a large-scale manner. The peripheral inflammation biomarkers could be evaluated by the blood cell counting of different leukocytes and the derived systemic inflammatory markers including neutrophil to lymphocyte ratio (NLR), platelet to lymphocyte (PLR), lymphocyte to monocyte ratio (LMR), and systemic immune-inflammation index (SII), which are cost-effective, convenient, and highly suitable for clinical applications (Grassano et al., 2023; Cao et al., 2023). These peripheral inflammation biomarkers have been used as predictive factors for the prognosis of cardiovascular diseases, autoimmune diseases, and neurodegenerative diseases like Parkinson's disease, Alzheimer's disease (Sharma et al., 2021; Stanca et al., 2022; Huang et al., 2023; Kuyumcu et al., 2012). As for ALS, a population-based study from Italy has found that increased neutrophils, NLR, and SII were associated with faster disease progression, worse pulmonary function and shorter survival (Grassano et al., 2023). Low lymphocytes and decreased LMR were correlated with poor prognosis in women ALS patients (Grassano et al., 2023). A retrospective, cross-sectional, observational study from China also demonstrated that NLR value was an independent parameter to predict the disease progression rate and survival in sporadic ALS patients (Wei et al., 2022). Among these systemic inflammatory markers, NLR is the most studied parameter in ALS, showing potential to predict ALS prognosis, while the evidences about the role of PLR, LMR, and SII are relatively rare (Cao et al., 2023; Wei et al., 2022; Choi et al., 2020; Leone et al., 2022). In addition, research data on ALS patients in China is still scarce compared with studies in the West.

Altogether, the aim of this study is to evaluate the role of the systemic inflammation status by analyzing NLR, PLR, LMR, and SII in Chinses ALS patients, to investigate if there were associations between these parameters with ALS severity, progression rate and survival. Thus, to determine the feasibility of these biomarkers as prognostic factors for ALS based on data from Chinese ALS patients.

## 2 Materials and methods

### 2.1 Patients

This retrospective study included patients who were diagnosed as definite, probable, possible, or suspected ALS in accordance with the revised El Escorial criteria at the Neurology Department of Nanjing First Hospital between January 2015 and June 2023. Patients classified as possible, or suspected ALS at the time of diagnosis were confirmed to be probable or definite ALS during follow-up. The ALS patients with the following conditions were excluded: (a) patients with incomplete baseline records; (b) presence of other fatal diseases despite of ALS; (c) patients with tracheostomy or receiving mechanical ventilation; (d) patients with percutaneous endoscopic gastrostomy. Eventually, a total of 72 ALS patients were included in the present study. Besides, 73 patients who came to Neurology Department of Nanjing First Hospital for physical examination were selected as controls. This study was approved by the Ethics Committee of Nanjing First Hospital in compliance with the Declaration of Helsinki revised in Brazil 2013. Both ALS patients and healthy controls gave their written informed consent.

### 2.2 Clinical data collection

Information about demographic features and disease-associated variables, including sex, age, BMI [calculated as weight (kg) divided by height (m)2], date of onset and diagnosis, diagnostic delay, disease duration, site of onset, phenotypes, and therapy (use of riluzole), were collected. The Amyotrophic Lateral Sclerosis Functional Rating Scale-Revised (ALSFRS-R) was used to evaluate the disease severity, with a total score of 48 point and higher scores indicate better functional state. The disease progression rate was calculated as monthly decline of the ALSFRS-R. The disease progression rate at diagnosis ΔFS = (48 – total ALSFRS-R at diagnosis)/disease duration in months; the disease progression rate after diagnosis ΔFS1 = (total ALSFRS-R at diagnosis – total ALSFRS-R at the last follow-up)/time interval between the initial and follow-up assessments.

### 2.3 Laboratory data collection and patient follow-up

Blood samples was drawn from the cubital vein of ALS patients and controls in the morning after overnight fasting at least 8 h, and measured in the hospital laboratory by standard methods within 1 h. We extracted the number of lymphocytes, neutrophil, monocyte and platelet from the medical files. NLR was calculated as the ratio of the neutrophil count to lymphocyte count, PLR was calculated as the ratio of the platelet count to lymphocyte count, LMR was calculated as lymphocyte to monocyte ratio, and SII was defined as (platelet × neutrophil)/lymphocyte. Telephone-based or face-to-face follow-ups were conducted every 6 months after the diagnosis until the occurrence of tracheostomy or death, or until December 2023 (the end of the follow-up).

### 2.4 Statistical analysis

Statistical analyses were performed with SPSS 22.0 (SPSS Inc., Chicago, IL, USA) and R statistical software version 4.4 (R Foundation, Vienna, Austria). The rate of disease progression was calculated as the change of Revised ALS Functional Rating Scale (ALSFRS-R) score per month. The histogram and density plots were used to show the distribution of disease progression rates of our patients and provide insight into whether a natural cutoff exists to stratified patients into fast and slow progressors. Continuous variables were expressed as mean ± standard deviation (SD), or median (interquartile range [IQR]) depending on whether they conformed to normal distribution. Categorical variables were expressed as frequency (percentage). Comparisons of continuous variables between two groups were analyzed by *t*-test or the Mann—Whitney *U*-test. The chi-square test was used to compare categorical variables. Log-transformed data were used for the analysis of continuous variables that did not conform to normal distribution. Spearman's correlation analysis was used to assess the association between NLR, PLR, LMR, SII and ALSFRS-R at diagnosis, and the association with ALS progression rate. Adjusted logistic regression analysis was performed to validate whether systemic inflammation markers were able to predict a faster disease progression rate in ALS patients. Kaplan-Meier curves, log-rank tests and multivariate COX proportional hazards regression models were applied to assess the effect of systemic inflammation markers on survival of ALS patients. In adjusted logistic regression models and multivariate COX proportional hazards regression models, each inflammatory biomarker was analyzed separately with the adjustments for covariates (including sex, age at onset, diagnostic delay, site of onset, BMI at diagnosis and use of riluzole). The cox.zph function from the survival package in R to test the proportional hazards assumption in the Cox models. The *p* value for each variable was > 0.05 in the cox.zph test, indicating that the proportional hazards assumption was not violated and the Cox model was appropriate in the present study. All *p*-values were two-sided, and a *p* < 0.05 was considered statistically significant.

## 3 Results

### 3.1 Demographic and clinical characteristics

A total of 72 ALS patients were finally analyzed in the present study, 42 men and 30 women. All patients were sporadic ALS without ALS family histories. The demographic and clinical features of ALS patients and healthy controls were summarized in Table 1. The mean age at onset was 60.37 (range 30–80 years), and the mean age at diagnosis was 61.39 (range 41–81 years), with median diagnostic delay of 10.19 months (ranging from 1 to 60 months). At the last follow-up, 37 (51.4%) patients had died. The mean disease durations for the dead and alive patients were 37.97 months and 27.15 months separately. Approximately 76 percent of the cases started with spinal symptoms, and the rest started with bulbar signs. The mean Amyotrophic Lateral Sclerosis Functional Rating Scale-Revised (ALSFRS-R) score at diagnosis was 38.67 (ranging from 18 to 48), and the median progression rate at diagnosis was 1.00 (IQR: 0.50–1.62, ranging from 0 to 5.6 points/month), the median progression rate after diagnosis was 1.03 (IQR: 0.59–1.50, ranging from 0 to 2.69 points/month). The age, sex, and BMI values were not significantly different between the healthy controls and ALS patients (all *p* > 0.05). Besides, the distributions of age and BMI were well-balanced across the ALS and control groups (Supplementary Figure S1). However, ALS patients had higher NLR, PLR, SII, and lower LMR values than health subjects (all *p* < 0.05).

**Table 1:** Clinical characteristics of ALS patients and health controls.

**Variable**	**Controls (*n* = 73)**	**ALS (*n* = 72)**	***p***
Gender-*N* (%)			0.667
Female	33 (45.2)	30 (41.7)	
Male	40 (54.8)	42 (58.3)	
Age (y)	59.07 ± 5.97	61.39 ± 9.86	0.090
Age at onset (y)		60.37 ± 10.02	
Diagnostic delay (m)		10.19 ± 10.70	
Disease duration-total (m)		32.86 ± 17.88	
Disease duration-dead (m)		37.97 ± 14.09	
Disease duration-alive (m)		27.15 ± 20.03	
Site of onset-*N* (%)			
Bulbar		17 (23.6)	
Spinal		55 (76.4)	
Escorial ALS-*N* (%)			
Definite		9 (12.5)	
Possible		24 (33.3)	
Probable		27 (37.5)	
Suspected		12 (16.7)	
BMI at diagnosis (Kg/m^2^)-*N* (%)	22.71 ± 2.93	22.58 ± 2.82	0.788
< 18.5	7 (9.5)	8 (11.1)	
18.5–24.9	56 (76.8)	55 (76.4)	
≥25	10 (13.7)	9 (12.5)	
Use of riluzole-*N* (%)			
Yes		42 (58.3)	
No		30 (41.7)	
ALSFRS-R		38.67 ± 7.08	
Progression rate at diagnosis		1.00 (0.50–1.62)	
Progression rate after diagnosis		1.03 (0.59–1.50)	
Death-*N* (%)			
Yes		37 (51.4%)	
No		35 (48.6%)	
NLR (Median [IQR])	1.58 (1.23–1.92)	1.94 (1.48–3.93)	< 0.001
PLR (Median [IQR])	101.05 (85.30–121.26)	128.46 (83.82–157.20)	0.003
LMR (Median [IQR])	4.91 (4.05–6.77)	3.54 (2.38–4.94)	< 0.001
SII (Median [IQR])	306.25 (231.79–409.04)	392.24 (247.23–753.13)	0.003

ALS, Amyotrophic lateral sclerosis; ALSFRS-R, Revised ALS Functional Rating Scale; NLR, neutrophil to lymphocyte ratio; PLR, platelet to lymphocyte; LMR, lymphocyte to monocyte ratio; SII, systemic immune-inflammation index (SII); IQR, Inter-Quartile Range; N, number; y, year; m, month.

### 3.2 Correlations between systemic inflammatory biomarkers and disease severity at admission in ALS patients

**Figure 1:**
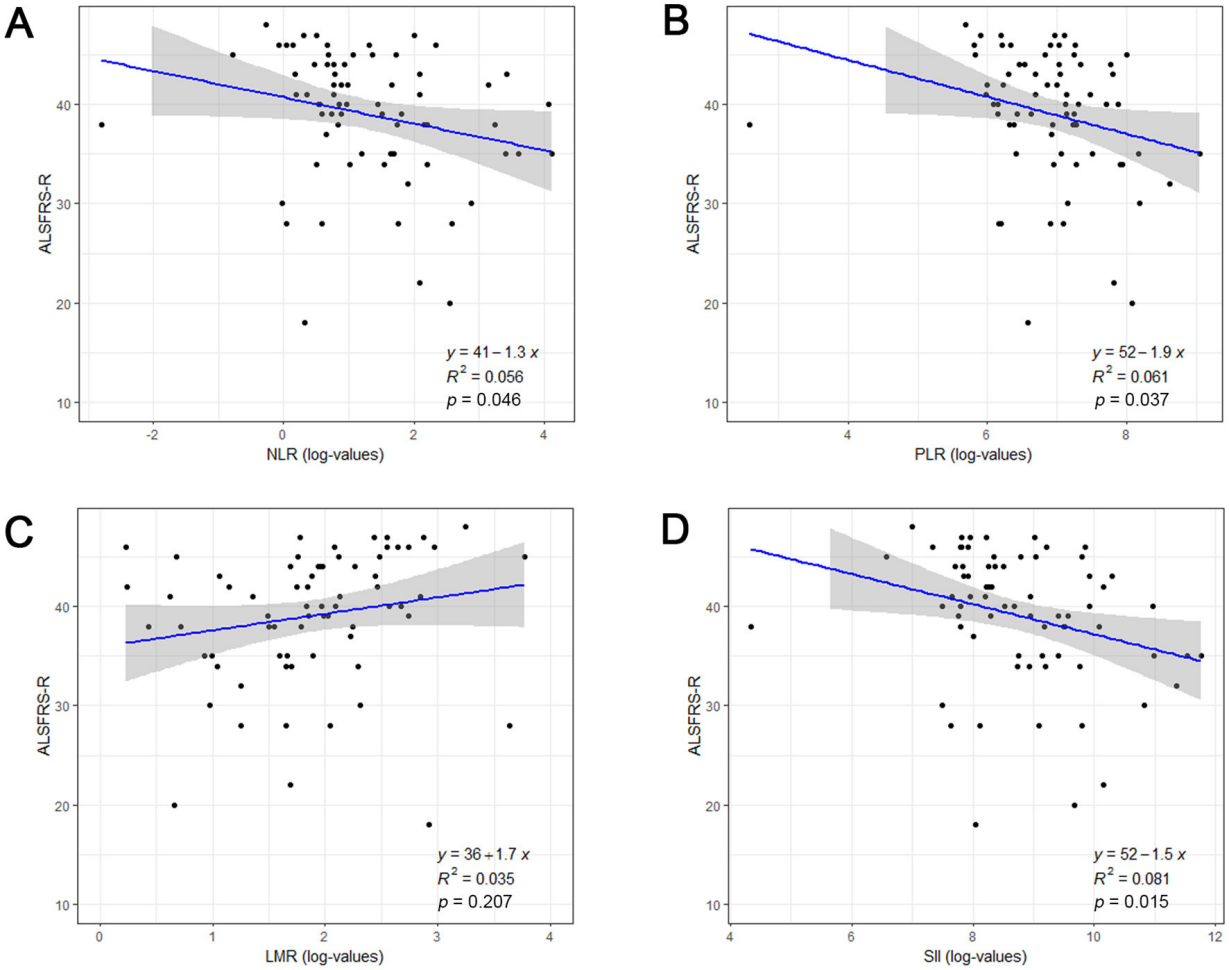
The association between systemic inflammatory biomarkers and disease severity at admission in ALS patients. Correlation between log-transformed NLR **(A)**, PLR **(B)**, LMR **(C)**, SII **(D)** and ALSFRS-R score. ALSFRS-R, Revised ALS Functional Rating Scale; NLR, neutrophil to lymphocyte ratio; PLR, platelet to lymphocyte; LMR, lymphocyte to monocyte ratio; SII, systemic immune-inflammation index (SII).

To assess the associations between systemic inflammatory biomarkers and disease severity, the ALSFRS-R was calculated in ALS patients at admission, with a total score of 48 point and higher scores indicate better functional state. As shown in Figure 1, NLR (*r* = −0.236, *p* = 0.046), PLR (*r* = −0.247, *p* = 0.037), and SII (*r* = −0.284, *p* = 0.015) were negatively correlated with ALSFRS-R, but not LMR (r = 0.151, *p* = 0.207). Above data indicated that ALS patients with higher NLR, PLR, or SII values might have poorer functional status upon admission.

### 3.3 Correlations between systemic inflammatory biomarkers and progression rate after diagnosis in ALS patients

**Figure 2:**
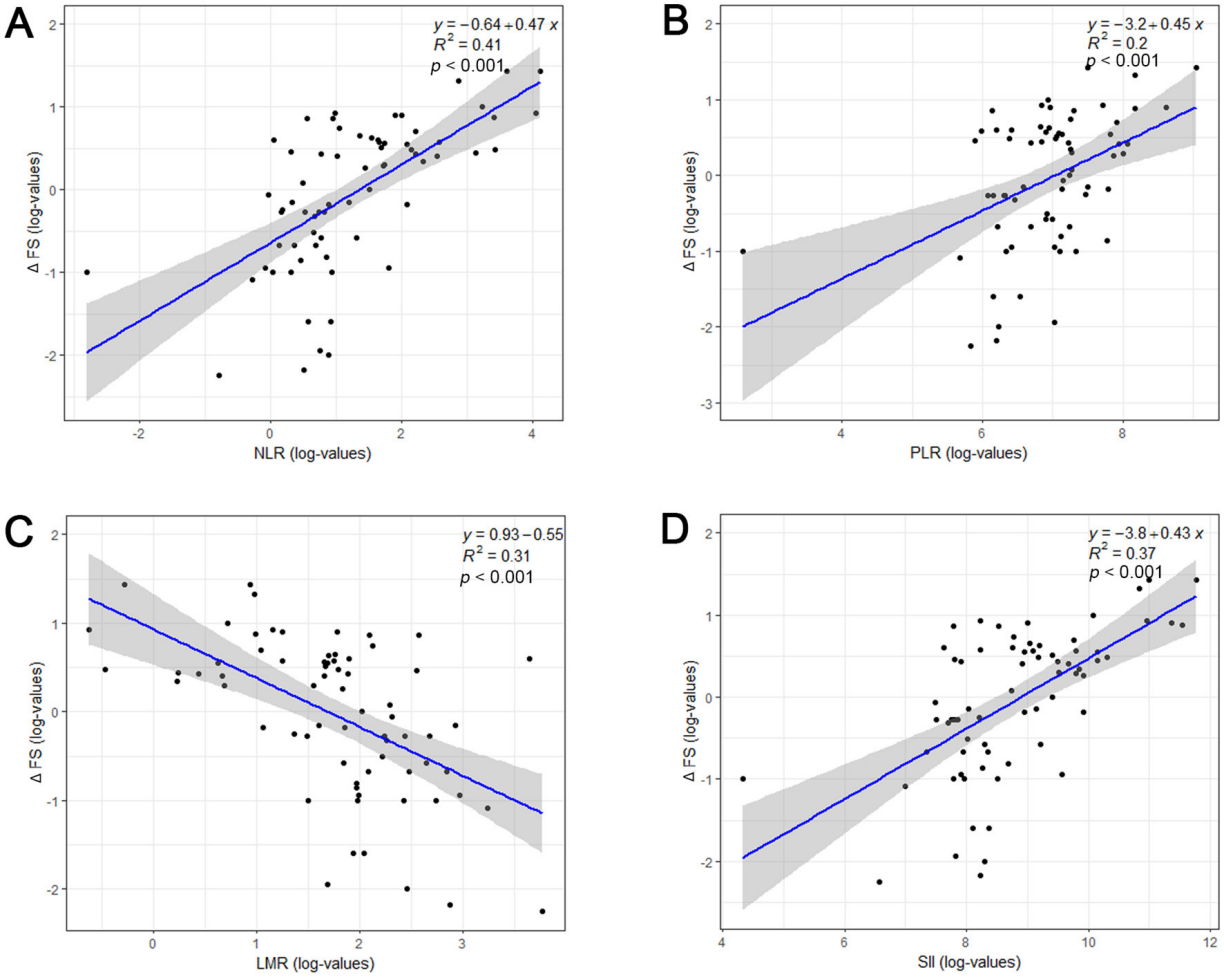
Relationship between systemic inflammatory biomarkers and disease progression rate (ΔFS) of ALS patients. Log-transformed NLR **(A)**, PLR **(B)**, LMR **(C)**, SII **(D)** and ΔFS values were shown by a scatterplot with fitted regression line, along with estimated Pearson correlation coefficient (*R*^2^) and *p*-value. ALSFRS-R, Revised ALS Functional Rating Scale; NLR, neutrophil to lymphocyte ratio; PLR, platelet to lymphocyte; LMR, lymphocyte to monocyte ratio; SII, systemic immune-inflammation index (SII).

Spearman's correlation analysis showed that NLR (*r* = 0.637, *p* < 0.001, Figure 2A), PLR (*r* = 0.444, *p* < 0.001, Figure 2B), and SII (*r* = 0.606, *p* < 0.001, Figure 2C) were positively correlated with disease progression rate after diagnosis, while LMR (*r* = −0.552, *p* < 0.001, Figure 2D) was negatively correlated with ALS progression rate. The distribution of ALSFRS-R decline rates was shown by the histogram and density plots (Supplementary Figure S2). From the distribution plots, it can be observed that the ALSFRS-R decline rates were primarily concentrated between 0.5 and 1.5, exhibiting a right-skewed distribution. A potential natural break point could be around a decline rate of 1.0, where the density plot shows a noticeable change in slope. Besides, both previous studies and clinical practice have consistently defined a decline of more than 1 point per month in the ALSFRS-R score as indicative of rapid progression, thus, patients were classified into fast progressors if the progression rate > 1.0 point/month and slow progressors if progression rate ≤ 1.0 point/month (Choi et al., 2020; Kimura et al., 2006; Czaplinski et al., 2006). Coincidentally, the patients were evenly divided into fast progressors and slow progressors, with 36 patients in each group. The detailed characteristics of these two subgroups patients were presented in Table 2. ALS patients with fast progression rate were older than patients. Significant higher percentage of bulbar signs at onset (*p* = 0.002), less use of riluzole (*p* < 0.001), and higher death rate (*p* < 0.001) were observed in ALS patients with fast progression rate than those with slow progression rate. NLR, PLR, and SII were remarkably higher, and LMR was significantly lower in fast progression group (all *p* ≤ 0.001, Table 2).

**Table 2:** Clinical characteristics of ALS patients with fast and slow progression rate.

**Characteristics**	**Fast (*n* = 36)**	**Slow (*n* = 36)**	***p***
Gender-*N* (%)			1.000
Male	21 (58.33)	21 (58.33)	
Female	15 (41.67)	15 (41.67)	
Age (y)	62.72 ± 8.56	57.47 ± 10.73	0.025
Age at diagnosis (y)	63.78 ± 8.67	58.67 ± 11.09	0.033
Diagnostic delay (m)	6.00 (3.25–9.75)	10.50 (5.25–12.00)	0.055
Education (years)	8.71 ± 3.86	9.50 ± 3.06	0.685
Site of onset-*N* (%)			0.002
Bulbar	14 (38.89)	3 (8.33)	
Spinal	22 (61.11)	33 (91.67)	
BMI at diagnosis	22.42 ± 3.02	22.74 ± 2.64	0.632
Use of riluzole-*N* (%)			< 0.001
Yes	13 (36.11)	30 (83.33)	
No	23 (63.89)	6 (16.67)	
ALSFRS-R	40.00 (35.00–43.00)	41.00 (39.00–44.00)	0.286
Progression rate at diagnosis	1.21 (0.56–2.09)	0.88 (0.38–1.47)	0.023
Death-*N* (%)			< 0.001
Yes	30 (83.33)	8 (22.22)	
No	6 (16.67)	28 (77.78)	
Disease duration-total (m)	31.86 ± 13.67	33.86 ± 21.43	0.770
NLR (Median [IQR])	3.55 (2.21–5.95)	1.60 (1.27–1.88)	< 0.001
PLR (Median [IQR])	144.17 (115.20–231.80)	111.17 (74.21–141.34)	0.004
LMR (Median [IQR])	3.04 (1.61–3.54)	4.30 (3.54–6.29)	< 0.001
SII (Median [IQR])	702.24 (434.96–1,125.33)	270.27 (221.93–359.46)	< 0.001

ALS, Amyotrophic lateral sclerosis; ALSFRS-R, Revised ALS Functional Rating Scale; ALS, Amyotrophic lateral sclerosis; ALSFRS-R, Revised ALS Functional Rating Scale; NLR, neutrophil to lymphocyte ratio; PLR, platelet to lymphocyte; LMR, lymphocyte to monocyte ratio; SII, systemic immune-inflammation index (SII); IQR, Inter-Quartile Range; N, number; y, year; m, month.

To further assess the association of systemic inflammatory biomarkers with ALS progression rate, univariable and multivariable logistic regression models were conducted. The univariable logistic regression model indicated that older age at onset, no use of riluzole, higher levels of NLR, PLR, SII, and lower level of LMR were associated with fast ALS progression rate (Table 3). The multivariable logistic regression models, which included sex, age at onset, diagnostic delay, site of onset, BMI at diagnosis, use of riluzole, demonstrated that faster disease progression was associated with higher NLR value (OR = 4.537, 95% CI = 1.904–10.815, *p* = 0.001), higher PLR value (OR = 1.016, 95% CI = 1.003–1.028, *p* = 0.013), lower LMR value (OR = 0.478, 95% CI = 0.299–0.765, *p* = 0.002), and higher SII value (OR = 1.005, 95% CI = 1.002–1.009, *p* = 0.001, Table 3).

**Table 3:** Logistic regression models for the association of NLR, PLR, and SII with disease progression rate in ALS patients.

**Variables**	**Unadjusted model**		**Adjusted model** ^*^	
	**OR (95% CI)**	***p***	**OR (95% CI)**	***p***
Sex	1.000 (0.392–2.552)	1.000	-	-
Age at onset	1.059 (1.006–1.114)	0.030	-	-
Diagnostic delay	0.969 (0.921–1.020)	0.230	-	-
Site of onset	1.167(0.393–3.467)	0.781		
BMI at diagnosis	0.960 (0.813–1.133)	0.626	-	-
Use of riluzole	0.113 (0.037–0.343)	< 0.001	-	-
**Inflammatory biomarkers**				
NLR	4.078 (2.004–8.301)	< 0.001	4.537 (1.904–10.815)	0.001
PLR	1.015 (1.005–1.025)	0.003	1.016 (1.003–1.028)	0.013
LMR	0.541 (0.381–0.768)	0.001	0.478 (0.299–0.765)	0.002
SII	1.005 (1.002–1.008)	< 0.001	1.005 (1.002–1.009)	0.001

ALS, Amyotrophic lateral sclerosis; ALSFRS-R, Revised ALS Functional Rating Scale; ALS, Amyotrophic lateral sclerosis; ALSFRS-R, Revised ALS Functional Rating Scale; NLR, neutrophil to lymphocyte ratio; PLR, platelet to lymphocyte; LMR, lymphocyte to monocyte ratio; SII, systemic immune-inflammation index (SII); IQR, Inter-Quartile Range; OR, odds ratio; CI, confidence interval. ^*^Adjusted for sex, age at onset, diagnostic delay, site of onset, BMI at diagnosis, use of riluzole.

### 3.4 Association of systemic inflammatory biomarkers and survival of ALS patients

**Figure 3:**
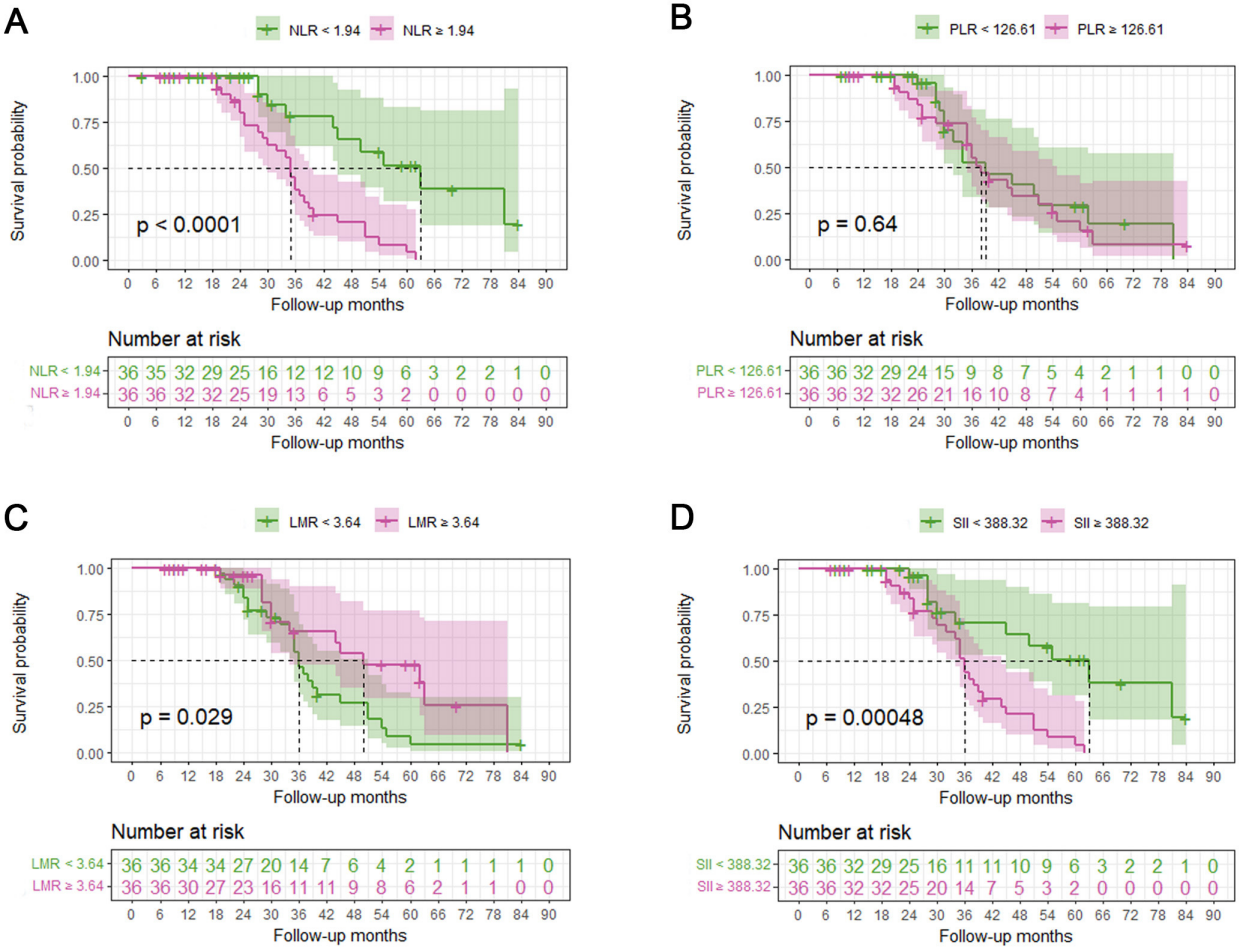
Relationship between systemic inflammatory biomarkers and overall survival of ALS patients. A median split was conducted on NLR, PLR, LMR and SII. Kaplan-Meier (KM) survival curves according to dichotomies of NLR **(A)**, PLR **(B)**, LMR **(C)**, and SII **(D)**. NLR, neutrophil to lymphocyte ratio; PLR, platelet to lymphocyte; LMR, lymphocyte to monocyte ratio; SII, systemic immune-inflammation index (SII).

Upon the completion of follow-up studies, 37 patients (51.4%) have died, 35 patients (48.6%) were alive. The mean survival time for all patients was 32.86 months, 37.97 months for those who had passed away, and 27.15 months for those who were alive. NLR, PLR, LMR, and SII were divided into two groups based on their median values. Kaplan-Meier analysis revealed that survival time was significantly shorter in groups with higher NLR (Log-rank, *p* < 0.001, Figure 3A), lower LMR (Log-rank, *p* = 0.029, Figure 3C), and higher SII (Log-rank, *p* < 0.001, Figure 3D). However, no significance was found between PLR and survival time (Log-rank, *p* = 0.64, Figure 3B).

Univariate and multivariate Cox proportional hazards regression analyses were used to explore the prognostic factors of survival. The univariate model showed that lower diagnostic delay, no use of riluzole, higher NLR, higher PLR, lower LMR, and higher SII values were associated with increased risk of mortality in patients with ALS (Table 4). After adjusting for sex, age at onset, diagnostic delay, site of onset, BMI at diagnosis, and use of riluzole, the continuous variable forms of NLR (HR = 1.321, 95% CI: 1.176–1.484, *p* < 0.001), PLR (HR = 1.005, 95% CI: 1.000-1.009, *p* = 0.001), LMR (HR = 0.803, 95% CI: 0.654–0.984, *p* = 0.035), and SII (HR = 1.001, 95% CI: 1.001-1.002, *p* < 0.001) represented potential as an independent parameter for survival prediction in the multivariate Cox regression analysis (Table 4).

**Table 4:** Univariate and multivariate Cox proportion hazards regression analyses for survival.

**Variables**	**Unadjusted model**		**Adjusted model** ^*^	
	**HR (95% CI)**	***p***	**HR (95% CI)**	***p***
Sex	0.814 (0.424–1.562)	0.535	-	-
Age at onset	1.011 (0.981–1.042)	0.484	-	-
Diagnostic delay	0.951 (0.913–0.990)	0.013	-	-
Site of onset	0.629 (0.291–1.361)	0.239		
BMI at diagnosis	0.962 (0.853–1.086)	0.534	-	-
Use of riluzole	0.422 (0.211–0.844)	0.015	-	-
**Inflammatory biomarkers**				
NLR	1.262 (1.150–1.385)	< 0.001	1.321 (1.176–1.484)	< 0.001
PLR	1.005 (1.001–1.010)	0.007	1.005 (1.000–1.009)	0.001
LMR	0.759 (0.636–0.906)	0.002	0.803 (0.654–0.984)	0.035
SII	1.001 (1.001–1.002)	< 0.001	1.001 (1.001–1.002)	< 0.001

ALS, Amyotrophic lateral sclerosis; ALSFRS-R, Revised ALS Functional Rating Scale; NLR, neutrophil to lymphocyte ratio; PLR, platelet to lymphocyte; LMR, lymphocyte to monocyte ratio; SII, systemic immune-inflammation index (SII); IQR, Inter-Quartile Range; HR, hazard ratio; CI, confidence interval. ^*^Adjusted for sex, age at onset, diagnostic delay, site of onset, BMI at diagnosis, use of riluzole.

## 4 Discussion

The systemic inflammation markers are easily available biomarkers derived from complete blood count and have been regarded as highly sensitive indicators of inflammation in several neurodegenerative diseases (Zhang et al., 2022; Jensen et al., 2021). However, the role of NLR, PLR, LMR, and SII in Chinese ALS has not been thoroughly studied. In the present study, we comprehensively evaluated the systemic inflammation markers of ALS patients who admitted to the Neurology Department of Nanjing First Hospital. Our results indicated that higher NLR, and lower LMR were associated with a faster disease progression rate and shorter survival time for Chinese ALS patients.

Numerous epidemiological studies have proposed both neuroinflammation and peripheral inflammation as relevant mechanisms in ALS. The peripheral inflammation could be represented by the blood cell counting of leukocytes, which are cost-effective, convenient, and highly suitable for clinical applications. Neutrophils, the most abundant type of WBC, represents the core of innate immunity and serves as fast-response cell in the immune system with phagocytosis and degranulation functions. Upon activation, neutrophils trigger inflammatory response through releasing oxygen-free radicals, lytic enzymes, and pro-inflammatory cytokines (Kolaczkowska and Kubes, 2013). Increased number of neutrophils in the peripheral blood has been demonstrated to be positively associated with ALS severity and fast rate of disease progression (Murdock et al., 2017, 2021). A recently published review comprehensively described the biological processes underlying neutrophil-induced pathogenesis in ALS (Cao and Fan, 2023). Lymphocytes is the core component of adaptive immunity. Different subtypes of T cells display different functions in ALS disease pathologies; T regulatory (Tregs) and CD4+ T cells appear to be protective, while CD8+ T cells appear to be destructive (Yazdani et al., 2022). As reported by Murdoc et al. and Beers et al., circulating CD4+ T cells and Tregs were significantly decreased in peripheral blood of ALS patients and inversely correlated with disease progression rate and severity (Murdock et al., 2017; Beers et al., 2017). The Tregs in ALS patients were dysfunctional, losing their ability to suppress responder T cell proliferation and proinflammatory macrophage/microglia cytokine synthesis and secretion (Beers et al., 2017; Sheean et al., 2018). These correlations have also been confirmed in mouse models, where passive transfer of wild-type CD4+ T lymphocytes into ALS mice lengthened disease duration and prolonged survival (Beers et al., 2011). Besides, a phase 2A double-blind, placebo-controlled Treg trial, which intravenously inject the expanded autologous Tregs into ALS patients, is currently in progress (Thonhoff et al., 2018; Appel et al., 2021). Monocytes and platelets also play important roles in the immune system (Menezes et al., 2009). The activate monocytes in peripheral circulation could secret IL-6 and TNF-α to aggravate disease symptoms and promote ALS progression (Zhao et al., 2017; Du et al., 2020). Detecting the changes of hematological markers in routine blood tests including neutrophils, lymphocytes, monocytes, and platelets, is the most generalizable and economical method to verify peripheral immunity sate. Furthermore, the four derived ratios including NLR, PLR, LMR, and SII have been regarded as novel biomarkers reflecting the balance between innate immunity and adaptive immunity and considered to be superior to the single parameters (Almǎşan et al., 2022; Qin et al., 2016). Thus, growing attention has been focused on these markers to reflect the degree of peripheral inflammation in neurodegeneration diseases (Zhang et al., 2022; Jensen et al., 2021).

Consistent with prior studies, NLR was also increased in ALS patients in the present study and negatively associated with disease severity, as defined by ALSFRS-R score (Grassano et al., 2023; Wei et al., 2022; Choi et al., 2020; Keizman et al., 2009). A remarkable correlation between increased NLR and the rapidly disease progression has also been observed in our study. Besides, we also detected significant increase of PLR, SII and decrease of LMR in ALS patients. However, previously reports did not report the significant association of PLR, LMR with ALS progression as the present study did (Grassano et al., 2023). Many differences exist in these markers depending on race, sex, and age. For example, the NLR value in the Asian population was generally lower than other races (Lee et al., 2018). The NLR was higher than 2 in all races except non-Hispanic black patients in the United State (Azab et al., 2014). The discrepancies between the present study and prior studies might be attributed to race and sample size differences. Larger sample size from multicenter are needed to verify the conclusions.

As reference values for NLR, PLR, LMR, and SII have not yet to be determined and many differences exist in these markers depending on race, sex, and age, the specific cut-off values of these systemic inflammatory markers for poor prognosis might not be generalizable to other ALS studies (Qin et al., 2016). So, the results of this study should be interpreted with caution. Besides, many studies have analyzed ALS prognosis according to the NLR tertiles (Wei et al., 2022; Choi et al., 2020; Leone et al., 2022), while the present study only averaged patients into two groups based on the median of NLR due to the limitation of small sample size. The cut-off values of the NLR for predicting the prognosis of ALS in our study was 1.94, which was lower than 2.315 reported in an Italian cohort (Leone et al., 2022). This result was consistent with previous finding that NLR in the Asian population was generally lower than that in Western countries (Leone et al., 2022). Besides, few studies have explored the cut-off values of PLR, LMR, and SII in ALS to predict poor outcomes. As discussed by previous researchers, the mechanisms and leukocytes involved in ALS peripheral immunity are diverse and multifaceted, and it is necessary to elucidate the complex mechanisms involved in ALS pathogenesis and progression through multiple markers instead of a single marker (Grassano et al., 2023).

There are some limitations in our study that needing future investigations. Firstly, this was a single-center study, which could add biases pertaining to the patient population and area healthcare practices. Secondly, the little sample size and *post hoc* nature of this study did not allow subgroup analyses by phenotype, genetic background, or other factors. The C9orf72 repeat expansion is a known risk factor for progression rate, but most of our patients did not perform genetic studies. However, C9orf72 repeat expansion is reported to be very rare in Chinese or Asian ALS patient (Zou et al., 2017). Besides, the follow-up time was short. Extending the follow-up time is needed to confirm the long-term significance of these systemic inflammatory markers. Lastly, we only measured the systemic inflammatory markers at a single time point during the course of disease. It's unknown whether dysregulation of the peripheral inflammation is a risk factor for ALS or a consequence of motor neuron degeneration. A recently published study assessed the longitudinal association between peripheral immune markers before disease onset and the incidence of ALS, and indicated that higher neutrophil counts, NLR, and SII were associated with an increased risk of incident ALS in a BMI- and Age-dependent manner (Cao et al., 2023). However, the causality needs to be supported by interventional experiments in both animal models and clinical trials.

In conclusion, our study explored the potential of baseline systemic inflammation markers as prognostic biomarkers for ALS patients. The results demonstrated that the easy-to-measure, available and inexpensive NLR, PLR, LMR, and SII might help to predict the disease progression rate and survival duration of sporadic ALS patients. Further multi-center studies with larger sample size, more varied populations, prospective designs, and long-term longitudinal measurements of the systemic inflammation markers are needed to validate the clinical potential of these markers.

## Data Availability

The original contributions presented in the study are included in the article/Supplementary material, further inquiries can be directed to the corresponding authors.

## Supplementary material

Supplementary Figure S1 The distribution of ALSFRS-R decline rates represented by histogram **(A)** and density plots **(B)**.

Supplementary Figure S2 Density plots to show the distribution of age and BMI for healthy controls **(A)** and ALS patients **(B)**. HC, healthy control; ALS, amyotrophic lateral sclerosis.
