# Supplementary figures and images for: The systemic inflammation markers as potential predictors of disease progression and survival time in amyotrophic lateral sclerosis
Source: Front Neurosci. 2025 Mar 5;19:1552949. doi: 10.3389/fnins.2025.1552949 (PMC11919871; doi:10.3389/fnins.2025.1552949)

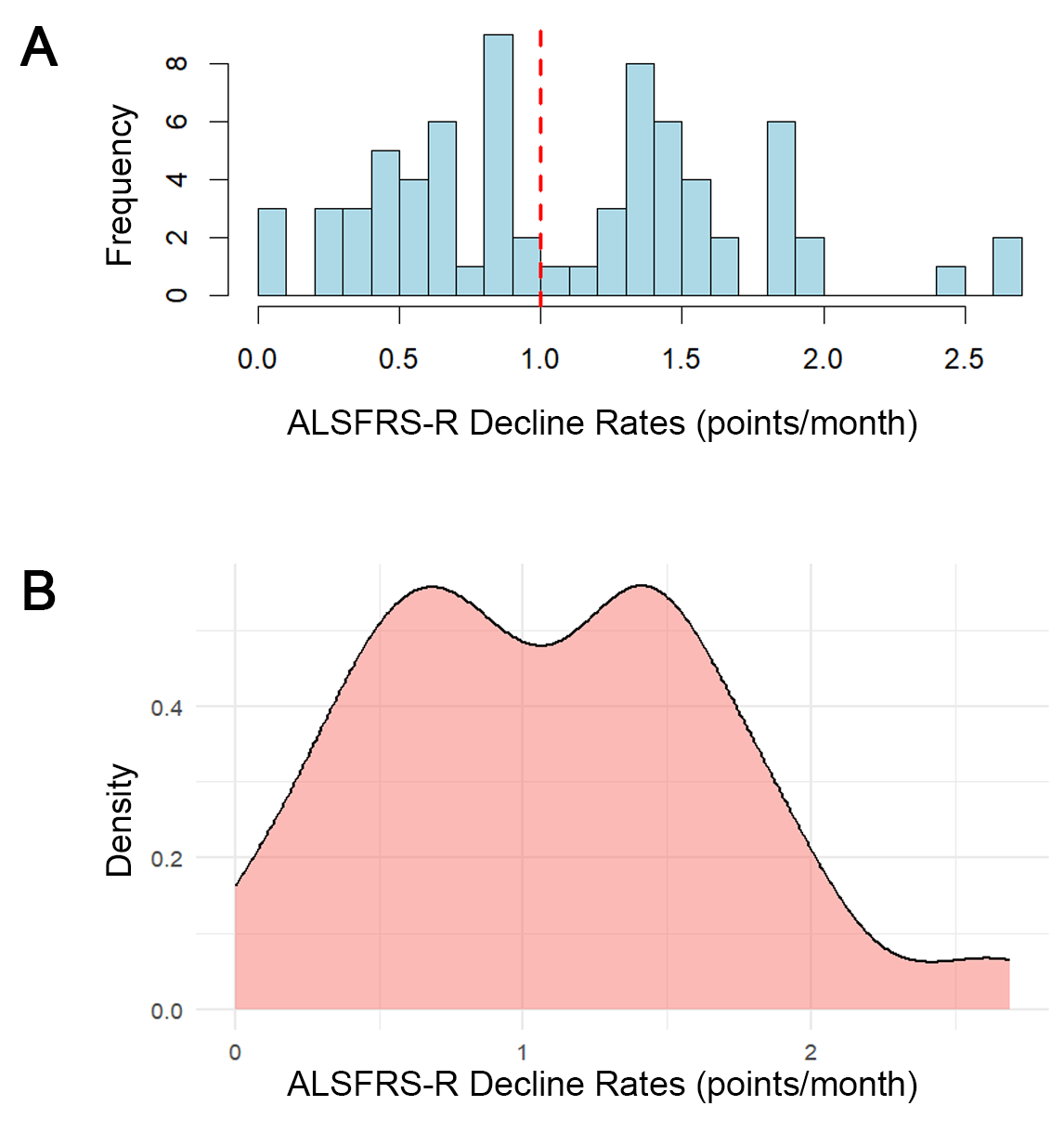

Supplement: Supplementary Figure S1 — The distribution of ALSFRS-R decline rates represented by histogram (A) and density plots (B). [file Image_1.tif]

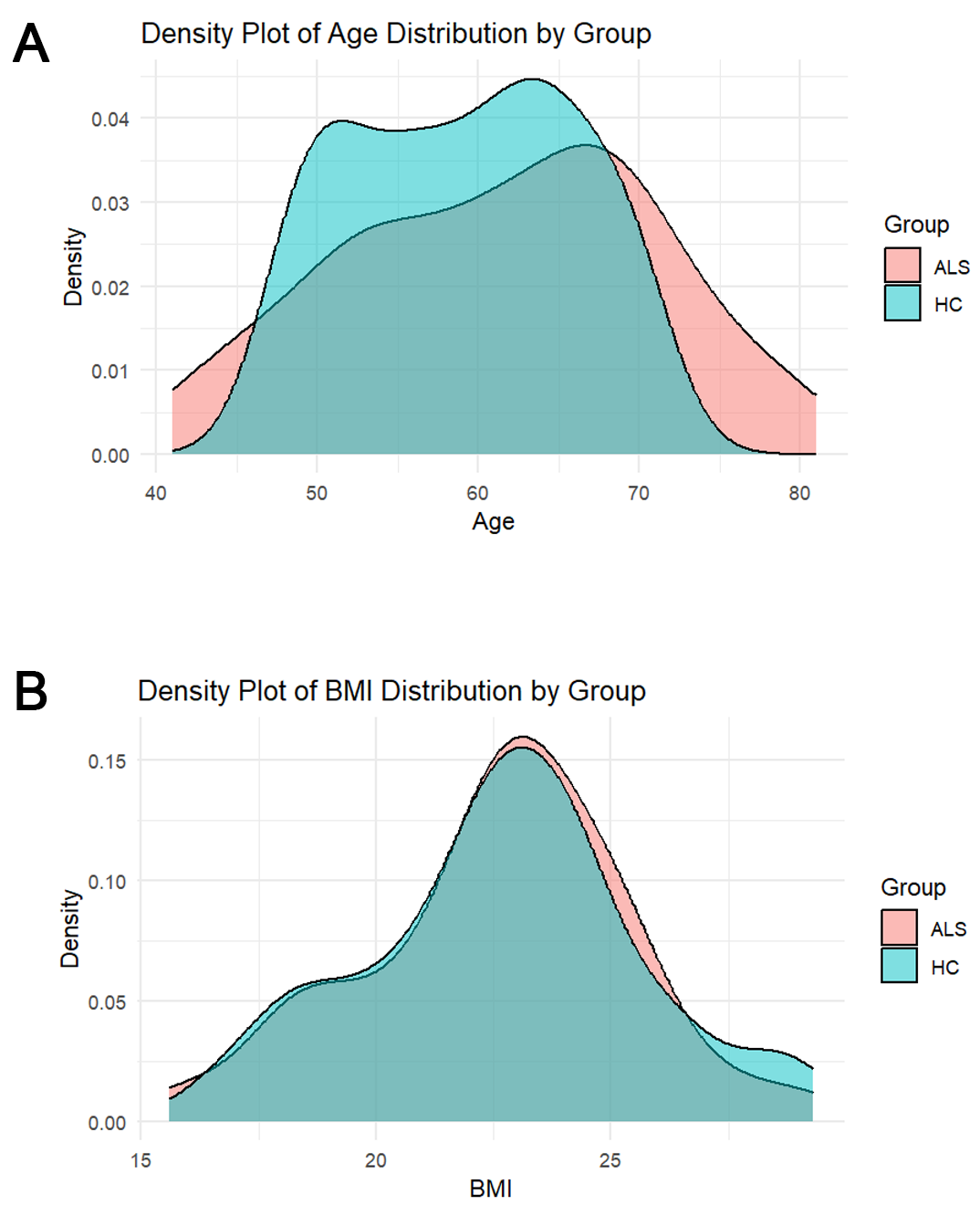

Supplement: Supplementary Figure S2 — Density plots to show the distribution of age and BMI for healthy controls (A) and ALS patients (B). HC, healthy control; ALS, amyotrophic lateral sclerosis. [file Image_2.tif]
